# Low-Cycle Fatigue Life Prediction of Titanium-Based Intermetallic Alloys Using Machine Learning and Finite Element Analysis

**DOI:** 10.3390/ma18081887

**Published:** 2025-04-21

**Authors:** Qiwen Xu, Guoqian Song, Xingwu Li, Yanju Wang, Aixue Sha, Yuanyuan Wei, Wenfeng Hao

**Affiliations:** 1 College of Mechanical Engineering, Yangzhou University, Yangzhou 225127, China; 2 Materials Evaluation Center for Aeronautical and Aeroengine Application, AECC Beijing Institute of Aeronautical Materials, Beijing 100095, China

**Keywords:** titanium alloys, Ti_2_AlNb-based alloys, fatigue damage, finite element, machine learning

## Abstract

This study explores the low-cycle fatigue characteristics of three structural components fabricated from Ti_2_AlNb-based alloys utilizing Seeger’s fatigue life theory and an improved Lemaitre damage evolution model. The validity and accuracy of the simulations based on these theoretical methods are verified by experimental fatigue life tests conducted at high temperatures. Additionally, the potential of employing long short-term memory (LSTM), extreme learning machine (ELM), and partial least squares (PLS) algorithms to predict the high-temperature, low-cycle fatigue life of Ti_2_AlNb alloy components is examined. Comparative analyses of the training effectiveness and practical applicability of these machine learning approaches are conducted, demonstrating that ELM exhibits superior predictive capability. This investigation thus provides a practical and efficient predictive methodology for assessing the low-cycle fatigue life of structural components composed of Ti_2_AlNb-based alloys.

## 1. Introduction

Ti_2_AlNb-based alloys, commonly termed O-phase alloys, were first identified by Banerjee et al. [1] in 1988 during their studies on heat treatments of Ti_3_Al alloys. They observed a newly stabilized orthorhombic phase (O phase) emerging due to the addition of Nb. Compared with TiAl-based alloys, Ti_2_AlNb alloys display enhanced room-temperature ductility, fracture toughness, and improved resistance to crack propagation. Furthermore, these alloys significantly reduce component weight compared with traditional nickel-based superalloys. Their outstanding properties at high temperatures enable prolonged service at temperatures ranging from 550 °C to 750 °C [2,3]. Consequently, Ti_2_AlNb alloys are increasingly adopted as lightweight, high-temperature structural materials in aeroengine components like casings and combustor liners. However, due to their sensitivity in terms of chemical composition and microstructure during processing, additive manufacturing (AM) methods offer superior control over traditional casting and forming processes in fabricating these alloys [4].

Ti_2_AlNb-based alloy is a lightweight, high-temperature-resistant intermetallic compound with three distinct phases: the β/B_2_ phase, α_2_ phase, and O phase. The constituent phases of these alloys can exist as a single phase, or two or three phases can coexist, depending on the heat treatment temperature and alloy composition. Zhou and Gussone et al. [5,6] investigated the phase transformation behavior of Ti_2_AlNb-based alloys under different solution heat treatment (SHT) and aging treatment (AT) temperatures. At 920 °C, the microstructure primarily consisted of lath-like O + α_2_ phases and discontinuous O + α_2_ phases at the grain boundaries. When the temperature increased to 1000 °C, needle-like O phases became dominant, and the tensile strength at 650 °C improved from 690 MPa to 820 MPa. However, above 1050 °C, the content of needle-like O phases decreased, and the B_2_ phase became the predominant constituent. During aging, the precipitation of O phases consumed a significant amount of Nb from the B_2_ matrix. With a prolonged aging time, the O phase tended toward saturation, leading to the formation of O/O phase interfaces, which act as crack initiation sites. Li and Zhang et al. [7,8] also reported that a substantial amount of needle-like O phases precipitate during aging in Ti_2_AlNb-based alloys, which contributes to enhanced tensile strength. Comparative studies under different heat treatment conditions demonstrated that the formation of a B_2_ matrix phase is beneficial for improving ductility and toughness, whereas the hexagonally close-packed α_2_ phase helps to suppress B_2_ grain coarsening. A higher fraction of the orthorhombic O phase was shown to enhance the alloy’s creep resistance and high-temperature strength [9]. Moreover, Ti_2_AlNb-based alloys generally exhibit high hardness (typically in the range of 400–500 HV). Zhang et al. [10] further found that the addition of Mo to Ti_2_AlNb-based alloys leads to the formation of well-aligned O + B_2_ microstructures. With appropriate solution and aging treatments, the hardness of Mo-modified Ti_2_AlNb-based alloys can reach up to 620 HV.

Bäumel and Seeger [11] first emphasized the necessity of employing different fatigue parameters for accurately predicting fatigue life in various metallic materials. They developed specific expressions for fatigue parameters for steel, titanium, and aluminum alloys based on Seeger’s generalized slope method. Yang et al. [12] further confirmed the accuracy of Seeger’s method through comparative analyses with other fatigue prediction approaches. Maliheh et al. [13] applied continuous damage mechanics (CDM) combined with Lemaitre’s ductile damage model to accurately forecast damage evolution in metals under different loading conditions. Liu et al. [14] integrated the Eshelby-Kroner localization principle into the Lemaitre fatigue damage model, introducing randomized parameters optimized through inverse analysis. Zhou et al. [15,16,17] proposed a refined Lemaitre damage evolution model with parameters specifically fitted for precise simulation of low-cycle fatigue damage in Ti-6Al-4V alloys.

Machine learning methods have increasingly been employed for fatigue behavior analysis and prediction in metallic materials [18]. Wang et al. [19] proposed a high-accuracy and high-stability, low-cycle fatigue life prediction method for Ti_2_AlNb-based alloy components based on continuum damage mechanics (CDM) theory and incorporating BP-ANN and GABP-ANN models. Heng et al. [20] introduced a novel hybrid neural network model combining convolutional neural networks (CNNs) with long short-term memory (LSTM) networks to predict fatigue life under both uniaxial and proportional multiaxial loads across different metallic materials. Duan et al. [21] compared several neural network models and identified LSTM as exhibiting superior predictive accuracy and generalization ability for high-cycle fatigue life in 304 stainless steel. The extreme learning machine (ELM), notable for its efficiency, simplicity, and robust generalization, has been widely applied across various fields [22]. Pan et al. [23] proposed a two-stage method based on ELMs for the fast and accurate prediction of the remaining service life of rolling bearings, demonstrating the ELM’s high accuracy and fast response capabilities even under small sample conditions. Duan et al. [24] compared support vector machines (nu-SVRs) and ELMs in predicting low-cycle fatigue life in 316 stainless steel, emphasizing the efficiency and effectiveness of ELMs.

Overall, research on the low-cycle fatigue life of open-hole structural components fabricated from Ti_2_AlNb-based alloys remains limited. This paper presents experimental investigations and fatigue life predictions for Ti_2_AlNb-based open-hole components under a stress ratio of 0.05, thereby addressing the current research gap. Additionally, preliminary calibration of fatigue parameters for Ti_2_AlNb-based alloys was performed, providing essential reference data for future studies. Finally, three conventional machine learning models were applied to predict fatigue life and analyze performance, offering a rapid and accurate method for predicting the fatigue life of titanium alloy structural components.

## 2. Methodology

### 2.1. Fatigue Life Prediction Theory

In this paper, Seeger’s fatigue life theory and the improved Lemaitre damage evolution model are employed to numerically predict the high-temperature, low-cycle fatigue life of Ti_2_AlNb-based alloys, further developing previous research [25,26]. The approximation formula proposed by Seeger for fatigue life prediction is presented as follows:

(1) $$2N_{f}={\left(\frac{\varepsilon_{f}^{\prime }E}{\sigma_{f}^{\prime }}\right)}^{\frac{1}{b-c}}$$

where b represents the fatigue strength exponent, typically in the range of (−0.05, −0.12), c denotes the fatigue ductility exponent, usually within the range of (−0.5, −0.7), $\sigma_{f}^{\prime }=1.67\sigma_{b}$, and $K^{\prime }=1.61\sigma_{b}$, with $\sigma_{b}$ defined as the ultimate tensile strength.

The cumulative damage per loading cycle, as described in the formulation of the improved Lemaitre damage evolution theory, is calculated as follows:

(2) $$\frac{\delta D}{\delta N}=\frac{{\left(\frac{∆\sigma_{eq}}{2}\right)}^{2+\frac{1}{n}}R_{V}}{ES{\left(K\right)}^{\frac{1}{n}}{\left(1-D\right)}^{\alpha +\frac{1}{n}}\left(1-\frac{D}{D_{c}}\right)\left(1+2n\right)}$$

The above equation describes the cumulative damage of Ti_2_AlNb-based alloy structural components during each loading cycle. In this expression, D is the damage variable, $D_{c}$ is the damage limit, which is set at 0.06, α represents the material constant, with a value of 4.1, K is the cyclic strengthening coefficient, valued at 2104, n is the cyclic strain-hardening exponent, equal to 0.133, and $∆\sigma_{eq}$ indicates the equivalent stress range.

### 2.2. Ti_2_AlNb-Based Alloy Material Properties

**Figure 1:**
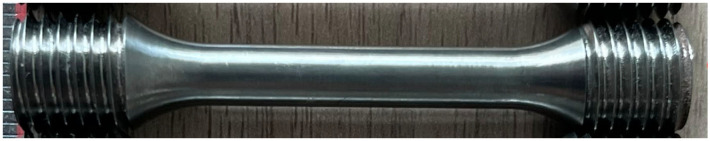
Smooth round-bar structural parts of Ti_2_AlNb-based alloy.

**Figure 2:**
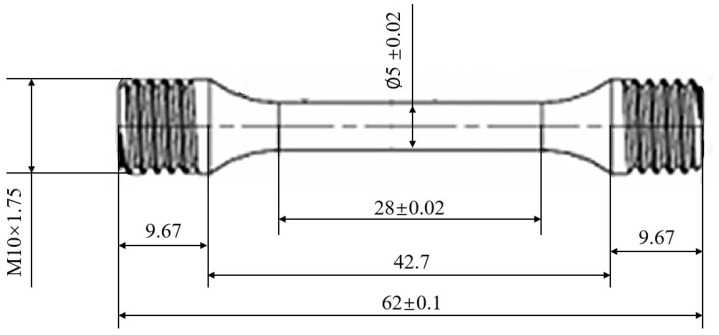
Dimensional drawing of smooth, round-bar specimen.

Smooth, round-bar structural parts of Ti_2_AlNb-based alloy, as shown in Figure 1 and Figure 2. were used to perform high-temperature tensile tests and acoustic resonance method tests (ASTM E1875-20) to determine the material parameters. The test temperatures were 550 °C and 650 °C, with strain rates of 0.001/s and 0.0001/s. The experimental results indicated that at 550 °C, the elastic modulus, Poisson’s ratio, and tensile strength were 102,560 MPa, 0.314, and 960 MPa, respectively. At 650 °C, these parameters were found to be 94,100 MPa, 0.316, and 847 MPa, respectively. Detailed results are summarized in Table 1.

### 2.3. Finite Element Simulation of Ti_2_AlNb-Based Alloy Structural Parts

**Figure 3:**
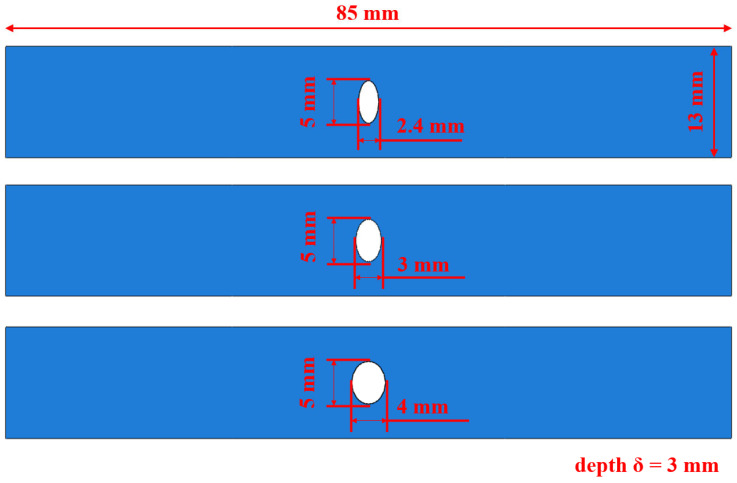
Dimensions of structural parts of three Ti_2_AlNb-based alloys.

Three representative elliptical open flat parts with a = 2.4, a = 3, and a = 4 were designed for the complex aero-engine casing, as illustrated in Figure 3. Finite element simulations were carried out using ABAQUS/Standard (The 2022 version). The finite element model was meshed with three-dimensional, four-node linear solid elements (C3D4). To ensure accurate representation of stress distributions under cyclic loading, a refined mesh convergence analysis was performed in the areas of interest.

Seeger’s fatigue life theory was applied by incorporating static tensile test data of Ti_2_AlNb-based alloy components into FE-SAFE for fatigue analysis. In these tests, a stress ratio of R = 0.05 was maintained, and the fatigue life was estimated using an empirical relationship. The mesh configuration included a global size of 1 mm, local refinement to 0.2 mm, and a transition mesh of 1 mm, enhancing the resolution in high-stress regions. The resulting fatigue life contours highlight variations in fatigue performance across the structure, with finer meshing at stress concentrators improving computational accuracy.

The modified Lemaitre damage evolution model was implemented via a user-defined subroutine in ABAQUS, allowing damage-induced stiffness degradation to be modeled through the output of solution-dependent state variables (SDVs). The damage threshold $D_{c}$ was set to 0.06 [17], beyond which material failure would be assumed and element deletion triggered. The corresponding number of cycles marks the predicted fatigue life. In this simulation, the mesh was set to 2 mm globally and 1 mm locally. Unlike Seeger’s approach, the finer mesh in damage simulations accelerates local stiffness loss, potentially leading to greater prediction errors due to early simulation termination near stress concentration zones.

### 2.4. Machine Learning Models and Fatigue Databases

The application of artificial neural networks in fatigue life studies of metal structural components shows significant potential. Their main strength lies in the ability to learn from experimental data and model both linear and nonlinear correlations within that data. In this paper, three machine learning models—the long short-term memory (LSTM) network, extreme learning machine (ELM), and partial least squares (PLS)—are utilized to train predictive models for the fatigue life of Ti_2_AlNb-based alloy components. The performance of these models is evaluated through high-temperature, low-cycle fatigue experiments, and their prediction results are comparatively analyzed.

Based on previous work [26], a total of 120 data sets from high-temperature, low-cycle fatigue tests and numerical simulations of Ti_2_AlNb-based alloy components were compiled. Among them, 58 datasets pertain specifically to the three structural configurations examined in this work. The model inputs consisted of factors influencing fatigue life, which exhibited a nonlinear relationship with fatigue behavior. These input variables included the component type, nominal stress, temperature, and stress concentration factor, while the output was the corresponding fatigue life of the Ti_2_AlNb-based alloy structural parts.

## 3. Testing and Finite Element Simulation of Ti_2_AlNb-Based Alloy Structural Parts

In this section, three representative structural components were designed based on the configuration and operational conditions of aeroengine combustor liners. Fatigue experiments were conducted at elevated temperatures of 550 °C and 650 °C under three typical loading conditions, which served as the baseline parameters. Subsequent high-temperature, low-cycle fatigue tests were carried out, and the fatigue performance of the components was further assessed using simulation approaches based on Seeger’s fatigue life model and a modified Lemaitre damage evolution framework.

### 3.1. High-Temperature Fatigue Test of Three Kinds of Open-Hole Structural Components

Three types of open-hole structural components were subjected to fatigue testing using a high-temperature fatigue testing system (Instron 8801, Instron Technologies LLP, Pimpri-Chinchwad, Maharashtra) operating in load-controlled mode. A triangular waveform was applied, with a stress ratio of 0.05 and a loading frequency of 10 Hz. Considering the actual service environment of Ti_2_AlNb-based alloy components, the test temperature was maintained at 550 °C, with peak stress levels set at 400 MPa, 576 MPa, and 700 MPa, respectively. Detailed experimental results are presented in Table 2.

### 3.2. Finite Element Simulation of Three Open-Hole Structural Components

**Figure 4:**
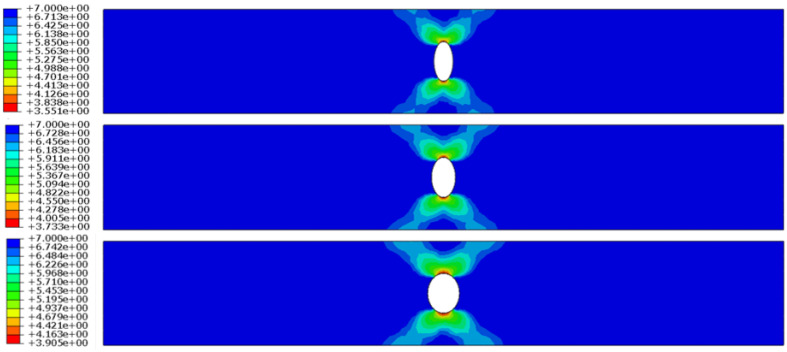
Fatigue life cloud using Seeger’s fatigue life theory.

**Figure 5:**
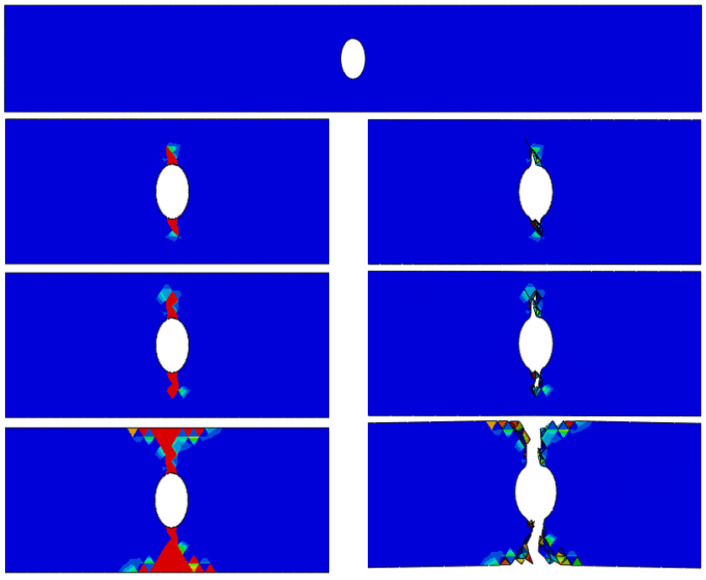
Damage evolution cloud for the a = 2.4 structural member (P = 400) under the improved Lemaitre damage evolution theory at 550 °C.

In the finite element analysis, simulation outcomes derived from Seeger’s fatigue life model and the enhanced Lemaitre damage evolution theory were compared against the average values of experimental results to validate the accuracy and reliability of both theoretical frameworks. At 550 °C, the deviations between the predicted fatigue life using Seeger’s theory and the experimental data were 11.7%, 6.7%, and 8.7%, respectively. Corresponding errors for the improved Lemaitre model were calculated to be 11%, 17.7%, and 9%. The fatigue life contour plots generated from Seeger’s model for the three structural components under a temperature of 550 °C and a peak stress of σ = 400 MPa are displayed in Figure 4. Figure 5 presents the damage evolution and failure contour plots based on the improved Lemaitre model, where the fracture contours illustrate the effect of element deletion upon reaching the critical damage threshold. Additionally, the fatigue life of Ti_2_AlNb-based alloy components at 650 °C was estimated using both theoretical approaches, and the results are summarized in Table 3.

## 4. Three Machine Learning Models

In this section, the high-temperature, low-cycle fatigue life of Ti_2_AlNb-based alloy structural components is predicted using a long short-term memory (LSTM) network, extreme learning machine (ELM), and partial least squares (PLS) algorithms. The prediction performance of the three machine models is also compared.

### 4.1. Long Short-Term Memory

**Figure 6:**
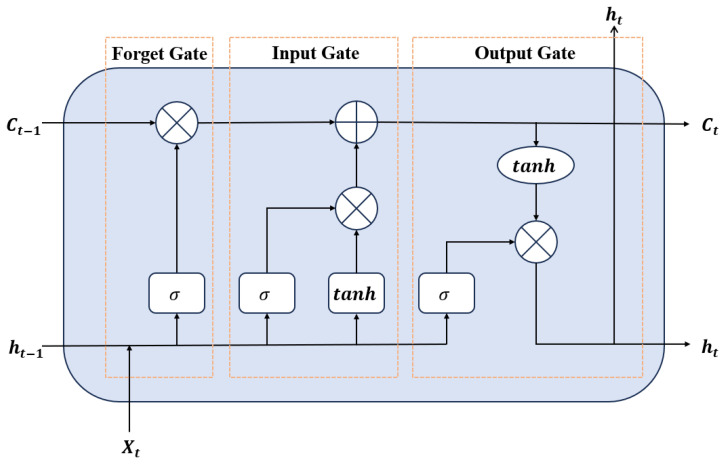
Schematic of the structure of LSTM.

The long short-term memory (LSTM) neural network is a refined version of the conventional recurrent neural network (RNN), developed to mitigate the limitations of standard RNNs in capturing long-term dependencies. Traditional RNNs, composed of basic recurrent units, struggle to maintain relevant information when the sequence length increases significantly. To address this challenge, Hochreiter et al. [27] introduced the LSTM architecture, which incorporates specialized components called “gates” within each memory cell. These include input, output, and forget gates, as well as a cell state acting as the hidden layer’s memory. The LSTM model framework adopted in this study is illustrated in Figure 6. The functionality of the three primary gates is described below.

The forget gate regulates which information from the previous cell state should be discarded. It is computed as follows:

(3) $$f_{t}=\sigma \left(W_{f}\left[h_{t-1},x_{t}\right]+b_{f}\right)$$

where $f_{t}$ is the forget gate’s output, σ denotes the sigmoid activation function, $W_{f}$ is the weight matrix associated with the forget gate, $h_{t-1}$ is the hidden state from the previous time step, $x_{t}$ is the current input, and $b_{f}$ is the bias term. The forget gate output is then element-wise multiplied with the previous cell state $C_{t-1}$, allowing the network to retain only relevant information.

The input gate controls which new information is added to the cell state. Its computation is defined as follows:

(4) $$i_{t}=\sigma \left(W_{i}\left[h_{t-1},x_{t}\right]+b_{i}\right)$$

(5) $${\tilde{c}}_{t}=\tanh\left(w_{\tilde{c}}\left[h_{t-1},x_{t}\right]\right.+b_{\tilde{c}})$$

where $i_{t}$ represents the gate’s output, while ${\tilde{c}}_{t}$ denotes the vector of candidate values to be added to the cell state.

The output gate controls which portion of the updated cell state will be exposed as the hidden state for the current time step and passed on to the next one. It plays a critical role in determining the network’s output at each stage in the sequence:

(6) $$o_{t}=\sigma \left(W_{o}\left[h_{t-1},x_{t}\right]+b_{o}\right)$$

(7) $$h_{t}=O_{t}\cdot \tanh\left(c_{t}\right)$$

where $o_{t}$ denotes the gate’s output, $h_{t}$ is the hidden state at the current time step, and $c_{t}$ is the cell state of the current time step.

The cell state $c_{t}$ is updated through a combination of the outputs from the forget and input gates, which together determine what information is discarded and what is newly stored:

(8) $$c_{t}=f_{t}\cdot C_{t-1}+i_{t}\cdot {\tilde{c}}_{t}$$

**Figure 7:**
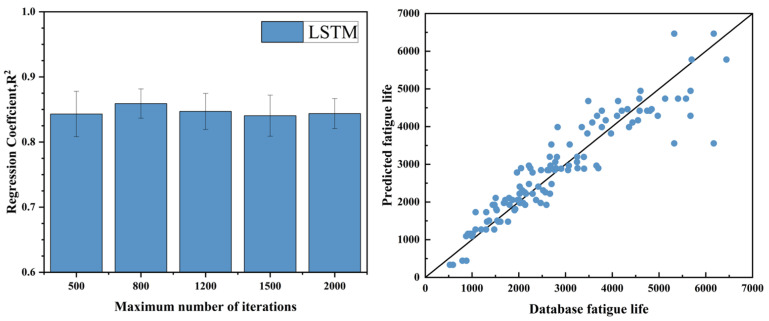
Hyperparameter optimization results of long-short term memory network and comparison of experimental and predicted lifetimes.

For training the LSTM model, the rectified linear unit (ReLU) activation function was employed in the activation layer, while the Adam optimizer was utilized to perform gradient-based optimization. Hyperparameter tuning is essential to the model’s effectiveness. The initial learning rate was set to 0.01, with a decay factor of 0.1 applied to reduce the learning rate as training progressed. To enhance data randomness and prevent overfitting, the randperm function was used to shuffle the dataset after each epoch. The number of training iterations is a critical hyperparameter. Five different maximum iteration values were tested. For each configuration, the model was trained 10 times, and the mean regression coefficient was calculated to assess performance consistency. The training outcomes are illustrated in Figure 7.

### 4.2. Extreme Learning Machine

The extreme learning machine (ELM) is a rapid and efficient training method for single hidden layer feedforward neural networks (SLFNs), originally introduced by Huang et al. [22] and extensively studied in later works. Unlike conventional learning approaches, the ELM achieves model training by simultaneously minimizing the training error and optimizing the output weights. This method significantly improves the training speed and generalization capability, making it highly suitable for applications such as fatigue life prediction and fault detection. The core steps of the ELM algorithm are summarized as follows.

The input training set is expressed by

(9) $$D=\left\{x_{i},\left.t_{i}\right|i=1,2,\ldots ,N\right\}$$

where $x_{i}$ is the input feature and $t_{i}$ is the label.

The SLFNs with activation functions and the number of hidden neurons are modeled as follows:

(10) $$\sum_{j=1}^{\tilde{N}}\beta_{i}g\left(W_{i}X_{j}+b_{j}\right)=O_{j},j=1,2,\ldots ,N$$

where $\tilde{N}$ represents the total number of hidden neurons and $g\left(x\right)$ is the activation function. The parameters $\beta_{i}$ and $W_{i}$ refer to the output weight and input weight of the i-th hidden neuron, respectively. Similarly, $b_{j}$ is the bias term associated with the output layer, and $O_{j}$ denotes the output of the j-th neuron. If the single hidden layer feedforward neural network (SLFN) is capable of perfectly fitting the training samples with zero error, then there exists a set of parameters $b_{j}$, $W_{i}$, and $\beta_{i}$ that satisfies the following equation:

(11) $$\sum_{j=1}^{\tilde{N}}\beta_{i}g\left(W_{i}X_{j}+b_{j}\right)=t_{j},j=1,2,\ldots ,N$$

The above relationship can be expressed as Hβ=T.

For ELM, W and b can be arbitrary values, and β can be calculated by the least squares solution:

(12) $$\left\|H\left(w_{1},\ldots ,w_{\hat{N}},b_{1},\ldots ,b_{\hat{N}}\right)\hat{\beta }-T\|=\underset{\beta }{\min}\|H\left(w_{1},\ldots ,w_{\hat{N}},b_{1},\ldots ,b_{\hat{N}}\right)\beta -T\right\|$$

The above equations can be completed using the Moor–Penrose (MP) generalized inverse:

(13) $$\hat{\beta }=H^{+}$$

where $H^{+}$ is the generalized inverse.

**Figure 8:**
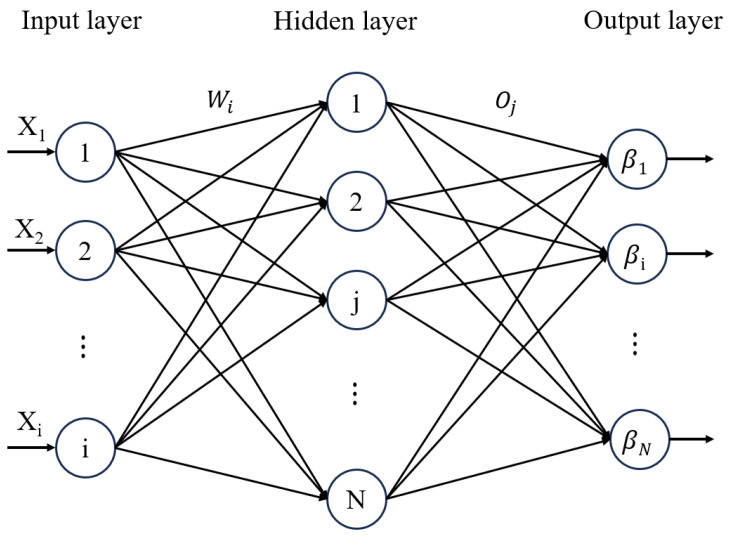
Schematic diagram of extreme learning machine (ELM) structure.

**Figure 9:**
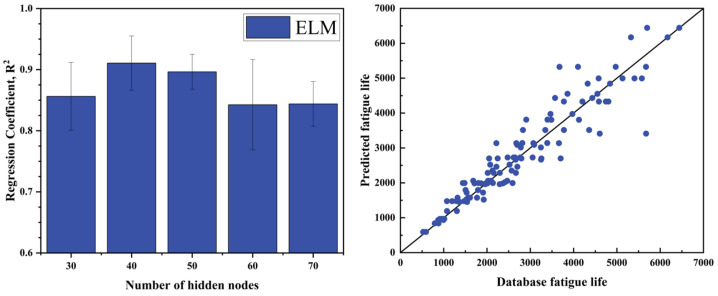
Extreme learning machine (ELM) hyperparameter optimization results and comparison of experimental life with predicted life.

In the ELM model, the sigmoid activation function is selected to map input data into the (0, 1) range, thereby embedding nonlinearity into the output response. An essential hyperparameter in ELM is the number of neurons in the hidden layer, which has a substantial impact on the model’s predictive performance. In this study, after evaluating multiple datasets, the optimal number of hidden layer nodes was determined to be 40. A schematic illustration of the ELM network architecture is provided in Figure 8, while the results of hyperparameter tuning and training performance are displayed in Figure 9.

### 4.3. Partial Least Squares Algorithm

**Figure 10:**
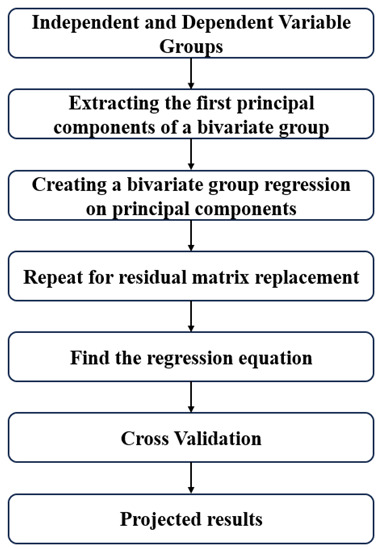
Flowchart of the partial least squares (PLS) algorithm.

The core principle of the partial least squares (PLS) algorithm is to construct a predictive model by identifying new orthogonal projection directions—referred to as principal components—that maximize the covariance between the independent and dependent variables in the projected space. Unlike many other machine learning methods, PLS can deliver reliable prediction results even when working with limited sample sizes. A simplified schematic of the PLS algorithm workflow is illustrated in Figure 10.

The first step involves normalizing both the independent and dependent variable matrices, followed by decomposition of the independent variable matrix:

(14) $$X=TP^{T}+E$$

Let X represent the matrix of independent variables, where T is the score matrix, P is the load matrix, and E is the residual matrix.

The matrix product $TP^{T}$ can be expressed as the sum of the products of the score vector $t_{j}$ and the load vector $p_{j}$, and thus we have

(15) $$X=\sum_{j=1}^{a}t_{j}p_{j}^{T}+E$$

The dependent variable matrix Y can be decomposed to

(16) $$Y=UQ^{T}+F$$

where U is the score matrix, Q is the load matrix, and F is the residual matrix, while $UQ^{T}$ can be expressed as the sum of the product of the score vector $u_{j}$ and the load vector $q_{j}$, as shown in the following equation:

(17) $$Y=\sum_{j=1}^{a}u_{j}q_{j}^{T}+\tilde{F}$$

Let $\hat{u_{j}}=b_{j}t_{j}$, where $b_{j}$ is the regression coefficient. Then, $\hat{U}=TB$, which ultimately yields $Y=TBQ^{T}+F.$

**Figure 11:**
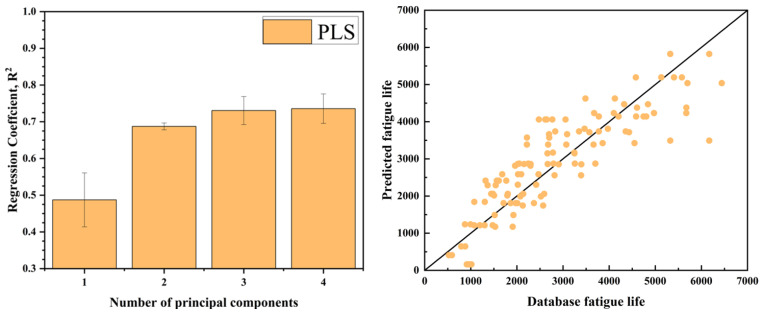
Partial least squares (PLS) algorithm hyperparameter optimization results and comparison of experimental life with predicted life.

PLS reduces the dimensionality of the features by extracting the principal components from the input data. The number of principal components retained determines the dimensionality of the feature space after reduction. Before performing PLS regression, the data are normalized. The optimization results, along with the experimental and predicted lifetimes, are shown in Figure 11.

### 4.4. Data Analysis and Discussion

In Section 3, high-temperature, low-cycle fatigue tests and finite element simulations of Ti_2_AlNb-based alloy structural components were conducted. By comparing the simulation data with experimental results, the errors between the fatigue life predictions from Seeger’s theory and the actual test data for three types of structural components (with a = 2.4, a = 3, and a = 4) were 11.7%, 6.7%, and 8.7%, respectively. The errors for predictions based on the improved Lemaitre damage evolution theory were 11%, 17.7%, and 9%, respectively. These errors fell within a reasonable range, demonstrating the accuracy and reliability of both theoretical methods. Therefore, these methods can be used to evaluate the fatigue performance of related components in future studies.

This study systematically investigated the influence of temperature, applied stress, and geometric characteristics on the high-temperature, low-cycle fatigue life of Ti_2_AlNb-based alloy structural components through controlled testing and simulation. According to Table 1 and Table 2, the fatigue life tended to decrease with rising temperatures and stress levels. Finite element simulations further showed that fatigue resistance diminished significantly during the final stages of the loading cycle, highlighting increased vulnerability to damage under prolonged loading. The stress concentration factor (kt) was used to quantify the intensity of localized stress within a structural component. For the three types of structural members with a = 2.4, a = 3, and a = 4, the stress concentration factors were 3.52, 3.06, and 2.55, respectively, as shown in Table 3. When the structural member had an elliptical opening, and the length of the axial opening was the same, the longer the length of the *x*-axis opening, the more relieved the stress concentration of the structural member was, and the more enhanced the ability to resist fatigue damage was.

In practical engineering applications, fatigue life assessment through physical testing and numerical simulation is often time-consuming and costly. Machine learning provides a promising alternative by enabling the learning of underlying physical relationships directly from data without requiring explicit prior knowledge. This makes it a valuable approach for predicting the fatigue life of titanium alloys. In this study, three machine learning techniques—long short-term memory (LSTM) networks, the extreme learning machine (ELM), and partial least squares (PLS) regression—were utilized to forecast the high-temperature, low-cycle fatigue life of Ti_2_AlNb-based alloy structural components. Each model underwent hyperparameter optimization to enhance performance, and the prediction outcomes are summarized in Table 4, Overall, the ELM model demonstrated the best performance among the three, with notable advantages in training speed, structural simplicity, and robustness. It achieved an average R^2^ of 0.892 and an RMSE of 0.177 across 10 runs, indicating high predictive accuracy. The LSTM model followed closely with an average R^2^ of 0.873, also showing strong predictive capability. In contrast, the PLS model yielded an average R^2^ of only 0.7357, suggesting limited effectiveness in capturing the nonlinear relationships inherent in the data.

The ELM model eliminates the need for iterative training by randomly assigning weights and biases between the input and hidden layers, distinguishing it from conventional machine learning approaches. This significantly enhances computational efficiency and the prediction speed. However, this study also found that improper selection of hyperparameters can lead to severe overfitting. The LSTM model possesses long-term memory capabilities and is well suited for handling complex data structures. In this study, a single-feature time step reconstruction approach was employed, treating each input feature as an independent time step. However, it was observed that the model required a large volume of training data to perform effectively, which may have contributed to its suboptimal predictive performance. The PLS model is appropriate for small-sample datasets, but it struggles with the highly complex and nonlinear nature of high-temperature fatigue behavior in titanium alloys. Its limited capacity to capture nonlinear relationships restricts its predictive performance in this context.

## 5. Conclusions

In this study, three representative structural components made of Ti_2_AlNb-based alloys were designed. Fatigue testing alongside numerical simulations was carried out at elevated temperatures of 550 °C and 650 °C, yielding satisfactory predictive accuracy. Based on the experimental and finite element method (FEM) results, three machine learning models—long short-term memory (LSTM) networks, the extreme learning machine (ELM), and partial least squares (PLS) regression—were applied to predict the fatigue life of the alloy components. These models demonstrated reliable and stable prediction performance while significantly reducing computation times. The findings offer valuable insights for fatigue life estimation of other alloys such as Ti-6Al-4V and TC4, especially for components with complex geometries. The primary conclusions of this work are as follows:

(1) In the finite element simulations of the three Ti_2_AlNb-based alloy structural components, Seeger’s fatigue life model produced prediction errors of 11.7%, 6.7%, and 8.7% compared with the experimental values, while the improved Lemaitre damage evolution model yielded errors of 11%, 17.7%, and 9%, respectively. These deviations are within an acceptable error margin, confirming the applicability of both models.

(2) The errors for the two fatigue life theories were 16.1%, 14.2%, and 17.4%, respectively. The reasonableness of both methods was validated by comparing the results with the experimental values.

(3) The impact of the hole geometry on fatigue life was clearly demonstrated. For the three elliptical hole structures investigated in this study, it was found that appropriately increasing the hole length in the x direction can effectively alleviate local stress concentrations, thereby enhancing fatigue life. Compared with conventional flat plates, incorporating surface structural reinforcement significantly improves the component’s resistance to fatigue failure.

(4) After hyperparameter optimization, the ELM model achieved the best prediction performance (R^2^ = 0.8919, RMSE = 0.17736), outperforming the other two models. The LSTM model ranked second, demonstrating strong scalability and the potential for improved performance with larger datasets. In contrast, the PLS model showed relatively weaker predictive capability and limited applicability. Overall, the ELM is more suitable for medium- and small-scale modeling tasks in the current study, while LSTM is better suited for complex scenarios involving strong temporal dependencies.

## Figures and Tables

**Table 1:** Mechanical parameters of materials.

Specimen Type	Temperature (°C)	Test Rate (s^−1^)	Tensile Strength (MPa)	Yield Strength (MPa)	Elongation at Fracture (%)	Young’s Modulus (GPa)
Smooth, round-bar specimen	550	0.001	961.5	820.1	36.2	99.400
	550	0.001	974.5	830.3	37.5	103.000
	550	0.001	941.5	770.9	15.8	109.000
	550	0.001	964.1	778.1	16.0	101.000
	550	0.001	968.2	811.3	15.8	102.000
	550	0.0001	961.3	847.1	18.4	97.100
	550	0.0001	962.3	832.0	14.8	101.000
	550	0.0001	946.1	805.3	16.7	108.000
	650	0.001	863.3	750.9	24.6	94.300
	650	0.001	863.3	756.7	39.5	92.000
	650	0.001	894.9	785.9	38.7	97.400
	650	0.0001	799.2	659.6	6.92	98.800
	650	0.0001	815.3	680.3	9.28	90.000
	650	0.0001	843.8	714.3	8.08	92.100

**Table 2:** High-temperature fatigue test results of three Ti_2_AlNb-based alloy structural components.

Specimen Type	Stress Concentration Factor (Kt)	Temperature (T) (°C)	Stress Ratio	Maximum Stress (MPa)	Nf-Real
a = 2.4	3.52	550	0.05	400	4806
					4204
					4747
				576	1711
					1864
					1975
				700	976
					985
					948
a = 3	3.06	550	0.05	400	5130
					5577
				576	2668
					2155
				700	1475
					1300
a = 4	2.55	550	0.05	400	7516
				576	2769
					2616
					2477
				700	1771
					1565
					1596

**Table 3:** Comparison of finite element simulation results of three Ti_2_AlNb-based alloy structural members.

Specimen Type	Temperature (T) (°C)	Stress (σ) (MPa)	Maximum Number of Failures			Two Kinds of Fatigue Theoretical Errors (%)
			Test Results (Average)	Seeger	Lemaitre	
a = 2.4	550	400	4586	4506	3776	16.1
		576	1850	2370	2009	
		700	1014	1014	914	
	650	400	/	2702	2219	
		576		996	874	
		700		592	526	
a = 3	550	400	5354	5407	4579	14.2
		576	2412	2296	2018	
		700	1388	1196	1075	
	650	400	/	4125	3486	
		576		1541	1365	
		700		880	795	
a = 4	550	400	7516	8036	7214	17.4
		576	2621	3054	2546	
		700	1644	1608	1321	
	650	400	/	6170	5326	
		576		2251	1967	
		700		1305	1076	

**Table 4:** Prediction performance results of three machine learning models.

Type of Method	$R^{2}$	RMSE	MAE	Time
Experiment	/	/	/	10–300 min
FEM	/	/	/	3–60 min
LSTM	0.873	0.19957	0.15418	10–15 s
ELM	0.8919	0.17736	0.17096	8–15 s
PLS	0.7357	0.19719	0.15261	5–10 s

## Data Availability

The original contributions presented in this study are included in the article. Further inquiries can be directed to the corresponding authors.
